# Cortical changes in patients with schizophrenia across two ethnic backgrounds

**DOI:** 10.1038/s41598-022-14914-3

**Published:** 2022-06-25

**Authors:** Benedikt P. Langenbach, Waldemar Kohl, Toshiya Murai, Thomas Suslow, Patricia Ohrmann, Jochen Bauer, Noriko Matsukawa, Shuraku Son, Anya Pedersen, Theresa Lichtenstein, Jun Miyata, Katja Koelkebeck

**Affiliations:** 1 LVR-Hospital Essen, Department of Psychiatry and Psychotherapy, Faculty of Medicine, University of Duisburg-Essen, Virchowstr. 174, 45147 Essen, Germany; 2 Center for Translational Neuro- and Behavioral Sciences, University Duisburg-Essen, Hufelandstrasse 55, 45147 Essen, Germany; 3 Department of Psychiatry and Psychotherapy, School of Medicine, University of Muenster, Albert-Schweitzer-Campus 1, Building A9, 48149 Muenster, Germany; 4 Department of Psychiatry, Graduate School of Medicine, University of Kyoto, 54 Shogoin-Kawahara-cho, Sakyo-ku, Kyoto, 606–8507 Japan; 5 Department of Psychosomatic Medicine and Psychotherapy, University of Leipzig, Semmelweisstraße 10, Building 13, 04103 Leipzig, Germany; 6 LWL-Hospital Muenster, Friedrich-Wilhelm-Weber-Straße 30, 48147 Muenster, Germany; 7 Department of Clinical Radiology, Medical Faculty, University Hospital Muenster, Albert-Schweitzer-Campus 1, Building A1, 48149 Muenster, Germany; 8 Department of Clinical Psychology, Faculty of Social Welfare, Hanazono University, 8-1 Nishinokyo Tsubonouchi-cho, Nakagyo-ku, Kyoto, 604-8456 Japan; 9 Clinical Psychology and Psychotherapy, University of Kiel, Olshausenstrasse 62, 24118 Kiel, Germany; 10 Department of Psychiatry and Psychotherapy, University of Cologne, Kerpener Strasse 62, 50934 Cologne, Germany

**Keywords:** Neuroscience, Brain

## Abstract

While it is known that cultural background influences the healthy brain, less is known about how it affects cortical changes in schizophrenia. Here, we tested whether schizophrenia differentially affected the brain in Japanese and German patients. In a sample of 155 patients with a diagnosis of schizophrenia and 191 healthy controls from Japan and Germany, we acquired 3 T-MRI of the brain. We subsequently compared cortical thickness and cortical surface area to identify whether differences between healthy controls and patients might be influenced by ethnicity. Additional analyses were performed to account for effects of duration of illness and medication. We found pronounced interactions between schizophrenia and cultural background in the cortical thickness of several areas, including the left inferior and middle temporal gyrus, as well as the right lateral occipital cortex. Regarding cortical surface area, interaction effects appeared in the insula and the occipital cortex, among others. Some of these brain areas are related to the expression of psychotic symptoms, which are known to differ across cultures. Our results indicate that cultural background impacts cortical structures in different ways, probably resulting in varying clinical manifestations, and call for the inclusion of more diverse samples in schizophrenia research.

## Introduction

Schizophrenia is known to be accompanied by pronounced neural changes in gray matter (GM). For example, patients with schizophrenia show GM reductions in the middle and inferior temporal gyrus, the anterior cingulate cortex, temporal cortex, parahippocampus, fusiform gyrus, insula and lingual gyrus^1,2^. Some changes, like GM reduction in the pars triangularis, seem to be a marker of the disease before its onset^3^, while GM reductions in the insula, thalamus, and prefrontal cortex seem to progress after the onset of the disorder.

However, as others have noted before, a separate analysis of both cortical thickness and surface area might yield a more nuanced view of structural brain changes, especially since they seem to be genetically and phenotypically independent^4^. The increased value of studying cortical thickness and surface area was not only shown for healthy processes like aging^5^, but also for schizophrenia, where cortical thickness and surface area might capture changes that are not identified by GM volumetric analyses alone^6^. Indeed, a number of changes in cortical thickness and surface area have been reported for schizophrenia, such as thinning in prefrontal and temporal areas^7–10^, and cortical changes seem to be rooted partly in both genetic and environmental factors^11–13^. Interestingly, changes may occur in either direction: for example, both cortical thickening and thinning have been observed in patients with schizophrenia^10^. To the best of our knowledge, it is still unclear whether cortical thickening is caused by the creation of new neurons or rather by pathological changes like intracellular edema. There is some evidence, however, that cortical changes represent a (insufficient) cortical reorganization of the brain after the onset of the disorder^14^. Thus changes in some areas might reflect a reaction to changes in others.

There is evidence that certain aspects of schizophrenia symptomatology differ depending on cultural context, e.g. alterations of social cognition (see ^15^ for an overview). Regarding delusions and hallucinations as core symptoms of schizophrenia, pronounced cultural differences have been reported: for example, religious delusions and delusions regarding poisoning seem to be more common in German than in Japanese patients, while the opposite holds true for delusions of reference related to harassment^16^. Similarly, there is evidence for lower rates of auditory and visual hallucinations in European compared to non-European schizophrenia patients^17^, and visual hallucinations are more common in West African patients than in European or patients from Pakistan^18^. However, visual hallucinations and auditory hallucinations seem to be more common in US-American patients than in Indian patients^19^. Even within Europe, there seem to be differences, as auditory hallucinations seem to be less common in Austria and Georgia compared to Poland and Lithuania^18^. Culture might not only affect the occurrence of specific types of hallucinations, but might also influence how other variables affect the disorder: for example, predictors like marital status differentially affected symptoms in US-American patients and Indian patients^19^. Regarding cognitive insight into the disorder, one study suggests that introspection and openness to feedback are closely related in Western samples, but separate entities in Indian patients^20^. While some of these results could be due to genetic differences, there is also evidence that the environment might have a stronger influence on the pathogenesis of delusions and hallucinations^21^. This type of research is of particular importance given the rather skewed nature of psychological, psychiatric and neuroscientific research that largely relies on “WEIRD” samples (Western, educated, industrialized, rich and democratic), even though “Western” countries only account for 12% of the world’s population^22–24^. More diverse samples would not only be beneficial for the understanding and treatment of mental disorders in non-Western populations. By comparing Western and non-Western samples, the influence of the cultural surroundings on the manifestation of mental disorders might be identified, which, in turn, might be beneficial for a deepened understanding, and ultimately better treatment, of these disorders regardless of cultural background.

 While more diverse samples are thus necessary, one should also be careful while using the concept of culture: notoriously ill-defined, the assumption of distinct and unchangeable cultures that are the sole explanation for psychological differences might in itself pose a problem and perpetuate problematic and stereotypical assumptions, for example when contrasting “the Western culture” with “the Eastern culture”^25^. When done carefully, however, the inclusion of culture might indeed help for a better understanding of the human psyche.

It is also well-known that brain functions related to social-cognitive and language abilities differ depending on the cultural context^26–28^.

Regarding broad cognitive markers such as cortical thickness and cortical surface area, pronounced differences have been found when comparing cross-cultural samples. For example, compared to (white) US-American participants, Korean participants had greater cortical thickness in areas such as the postcentral gyrus and the bilateral inferior temporal gyrus. US-Americans, on the other hand, showed more cortical thickness in the left transversetemporal cortex, lingual gyrus, and right lateral occipital cortex^29^. Interestingly, there also seems to be evidence for the fact that differences between international samples are age-dependent^30^. Consistent with the view that the cultural context affects brain plasticity^30^, different neural manifestations of mental disorders, depending on cultural context, are to be expected. However, empirical evidence for such differences in neural markers are scarce for schizophrenia: Gong et al.^31^, who studied Chinese, Japanese, African-Caribbean and White Caucasian patients with schizophrenia vs. healthy controls, found a consistent pattern of right anterior insula volume reductions independent of ethnicity in patients. In another study with differing ethnicities in the United Kingdom, no differences in GM volume loss were found across African-Caribbean and Black African patients as compared to White Caucasian patients^32^. Recently, different patterns of gray matter reduction were shown for Japanese and German patients with schizophrenia^33^.

To this date, however, no data is available on how cultural differences affect changes in cortical thickness and surface area in schizophrenia. However, not only are cortical thickness and surface area more sensitive measures than GM volume alone^6^, cortical thickness is also a known marker for severity of psychotic symptoms^7,8,10^.

In the study at hand, we address this research gap by reporting findings of two large samples from Japan and Germany comprising patients with schizophrenia as well as healthy controls. We investigated whether cortical thickness and surface area are differently affected in schizophrenia depending on the cultural context. While Germany and Japan are both industrialized, rich and democratic countries, there are also pronounced differences, for example regarding religious beliefs, cultural traditions, social norms or population density. Additionally, therapeutic interventions like medication and the nature of psychotherapeutic treatment show cultural specificities^34^.

Focusing, for the first time, explicitly on the differences in cortical GM thickness and surface area in patients with schizophrenia across two large culturally diverse samples might allow new insights into how the brain is affected by schizophrenia.

## Materials and methods

### Sample

In this study, a large sample of patients with schizophrenia was investigated from two study sites (Japan and Germany) acquired over the course of ten years (2005–2015). Structural imaging data were taken from participants recruited as part of single-center cross-sectional investigation projects at the respective sites. Healthy controls were matched by age, gender and education. The dataset included a total of 401 subjects (190 patients, 211 healthy controls). From Germany, 180 datasets (94 patients with schizophrenia and 86 healthy controls) were included in this study. From Japan, 221 datasets (96 patients with schizophrenia and 125 healthy controls) were analyzed. All patients were diagnosed with schizophrenia, schizoaffective disorder or schizophreniform disorder according to interviews performed with the Structured Clinical Interview for DSM-IV Axis I Disorders (SCID-I)^35^ by experienced interviewers. The samples included in- and outpatients and were mainly recruited from patients treated in the participating center at the time of the respective study. Healthy controls were recruited locally via advertisements and personal contacts as matched to the patient groups according to age, sex and education. All participants met the usual exclusion criteria, including severe neurological or internal medical disorders, any other current mental disorder or acute drug or alcohol abuse or dependence. After removing double entries, participants with gross anatomical abnormalities, corrupted data sets and one control participant with a post-hoc psychiatric diagnosis, 155 patients with schizophrenia and 192 healthy controls from both sites were included in the final sample. The specific schizophrenia subtypes were recorded for 110 of the 155 patients (67 of which were diagnosed with paranoid schizophrenia, see supplementary material for details). It should be noted, however, that long-term stability of schizophrenia subtypes is low^36,37^, and no further analysis of the sometimes small subgroups were calculated.

Clinical data which were assessed for both sites included chlorpromazine (CPZ) equivalents and duration of illness. Psychopathology was mainly assessed with the Positive and Negative Syndrome Scale (PANSS)^38^. In one German subset with 35 patients, however, the Scale for the Assessment of Positive Symptoms (SAPS)^39^ and the Scale for the Assessment of Negative Symptoms (SANS)^40^ were used to assess psychopathology. For comparison of the data, the method by van Erp et al.^41^ was used to transform SANS/SAPS to PANSS data. Handedness was measured with the Neurological Soft Signs (NSS)^42^ and the Edinburgh Handedness Scale^43^. Years of education were additionally assessed. Clinical and sociodemographic characteristics of the sample are compiled in Table 1.

**Table 1:** Summary of social and clinical data of the two samples.

	Japan		Germany	
	Patients	Controls	Patients	Controls
Sample size	81	111	74	81
Females	39	50	32	29
Mean age (SD) in years	37.4 (9.30)	32.1 (11.0)	29.1 (7.49)	30.4 (8.54)
Duration of illness (SD) in months	158 (102)		61 (67)	
Years of education	13.9 (2.06)	15.0 (2.71)	11.9 (1.53)	12.1 (1.35)
Handedness (right:left:other:NA)	(65:4:3:9)	(95:5:7:4)	(67:7:0:0)	(74:7:0:0)
CPZ (SD)	597 (716)		819 (716)	
PANSS +	14.3 (4.58)		13.3 (4.77)	
PANSS–	15.8 (5.28)		17.7 (5.51)	

CPZ = chlorpromazine equivalents; PANSS +  = Positive and Negative Syndrome Scale, Positive Scale; PANSS-: Positive and Negative Syndrome Scale, Negative Scale.

A one-way between-participants ANOVA revealed a statistically significant lower use of medication (standardized to CPZ) in the Japanese patients, F(1,152) = 5.39, *p* = 0.021. (One patient had an implausibly high medication dose and was thus excluded from all analyses which included CPZ equivalents). Additionally, Japanese patients had a longer average duration of illness (158 months vs. 61 months in the German sample) and Welch’s *t*-test revealed this difference to be statistically significant, t(138) = − 6.99, *p* < 0.001.

Ethical approval for all studies was granted by the local ethics committees, i.e. the Ethics Committee of the University of Muenster and the Westphalian State Chamber of Physicians as well as the Committee on Medical Ethics of Kyoto University. All conformed to the Declaration of Helsinki (https://www.wma.net/policies-post/wma-declaration-of-helsinki-ethical-principles-for-medical-research-involving-human-subjects, last access: 13/04/2022). All participants provided informed consent to participate in this study.

### MRI acquisition and processing

#### Image acquisition

Magnetic resonance imaging was performed on a Philips 3 T scanner (Gyroscan Intera 3 T, Philips Medical Systems, Best, The Netherlands) in Germany (repetition time (TR) = 7.5 ms, echo time (TE) = 3.4 ms, field of view (FOV) = 256 × 204, in-plane-matrix = 256 × 204, 160 slices à 1 mm, reconstructed voxel size 0.5 mm isotropic, 3DT1TFE-sequence). In Japan, a Siemens 3 T scanner (Trio, Siemens, Erlangen, Germany) was used to perform the scans (TR = 2,000 ms; TE = 4.38 ms; FOV = 225 × 240 mm, matrix = 240 × 256; resolution 0.9375 × 0.9375 × 1.0 mm^3^, inversion time (TI) = 990 ms, 3DMPRAGE-sequence).

#### Image processing

Cortical reconstruction and volumetric segmentation were performed with the FreeSurfer image analysis suite (all respective analyses were conducted on the same computer at Kyoto University, Japan), which is documented and freely available for download online *(*version 4.5.0; http://surfer.nmr.mgh.harvard.edu/*)*. To calculate thickness and surface area of the cerebral cortex, the 3D-MPRAGE images were used. This includes a Talairach transformation of each participant’s native brain, as well as removal of non-brain tissue and segmentation of GM and white matter (WM) tissue. The GM/WM boundary was tessellated to generate multiple vertices across the whole brain. The cortical surface of each hemisphere was inflated to an average spherical surface to locate the pial surface and the GM/WM boundary. Any topological defects were corrected manually after visually inspecting the cortex of each participant (blinded to identity). After cortical representations were created, neuroanatomical labels were assigned on a cortical surface model based on the automated labeling system, and the entire cortex was parcellated into 33 brain regions per hemisphere^44^. Cortical thickness was computed as the shortest distance between the pial surface and the GM/WM boundary at each vertex across the cortical mantle. The cortical volume was defined by surface-based volumetric calculation (area x thickness). The surface area of a region was computed by adding up the area of the vertices in that region^45^. The GM cortical volume (in mm^3^), mean thickness (in mm) and surface area (in mm^2^) were calculated for each brain region for both hemispheres separately.

### Statistical analyses

All statistical analyses were conducted using the statistical computing software R, version 4.0.0^46^, and SPSS, version 21. A linear regression analysis following the GLM was calculated for each cortex area, separately for the left and the right hemisphere, with either cortical thickness or surface area as the dependent variable: group (schizophrenia vs. control), site (Japan vs. Germany) and the interaction thereof as predictors. In linear regressions, an interaction term allows to investigate whether the influence of one independent variable (e.g., group) on the dependent variable (e.g., cortical thickness) changes depending on the value of another independent variable (e.g., site). Age and gender were inserted as covariates. Additionally, we controlled for overall cortical thickness/cortical surface area of the respective hemisphere (as is standard in comparisons of cortical thickness and cortical surface) to make sure that any effects were due to specific changes in the respective areas, not due to global differences in cortical thickness. By analyzing interaction effects between site (Japan vs. Germany) and group (patients vs. controls) it was possible to reduce the confounding effects of the magnetic resonance tomography (MRI) scanner to a minimum, as analyses rely on the relative differences between the group at each site. All p-values were adjusted for multiple comparisons in the respective hemisphere using the false discovery rate method^47^.

As explained in the description of our sample, an ANOVA was used to test for group differences regarding the duration of illness and medication between the German and the Japanese patients. In a next step, linear regressions were calculated (separately for each hemisphere), again using cortical thickness or surface area as dependent variable and either duration of illness or medication as predictors. Age, gender and overall cortical thickness/cortical surface area were used as covariates. This approach allowed us to investigate whether duration of illness and medication were correlated with the cortical measures. Finally, we analyzed whether there was a relationship between psychopathology (as assessed by the PANSS) and cortical thickness or cortical surface area, again using linear regression models with the same covariates.

For each regression and participant, Cook’s distance was calculated as a way of identifying potential outliers. Previously, it has been suggested that Cook’s distance of more than 1 indicates a potential outlier^44^. All values stayed below 1 (largest value: 0.51), indicating that no single participant had an excessive influence on the regression^48^. Still, after calculating the initial regressions, the results were checked for extreme outliers in the areas with statistically significant results and, if necessary, the calculations were rerun without outliers.

## Results

### Grey matter cortical thickness

**Figure 1:**
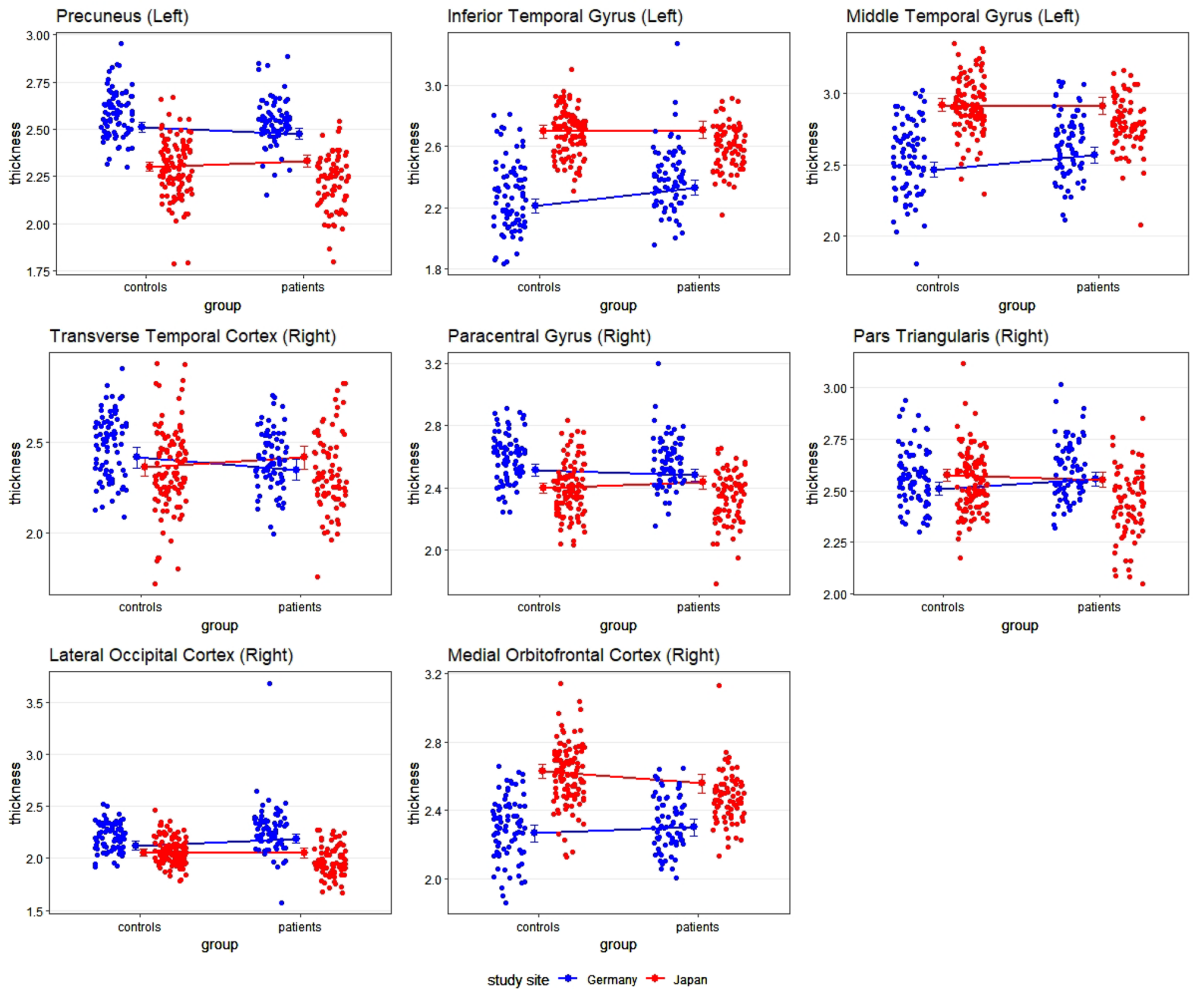
Scatterplots depicting the interaction between group and site with cortical thickness as dependent variable. The small dots represent the individual values per participant; the lines connect the estimated means (i.e., the output of the regression). Error bars indicate the 99% confidence interval.

There were multiple cortical areas in which an interaction between group (schizophrenia vs. controls) and site (Japan vs. Germany) was present. The statistics of the statistically significant interaction terms are presented in the supplementary material; a graphical depiction is shown in Fig. 1. For some areas, the interaction was driven by an effect of group (schizophrenia) only at one site. For example, no cortical changes were visible in the left inferior and middle temporal cortex and the right lateral occipital cortex in the Japanese sample, while there was a pronounced cortical thickening in German patients. In other areas, findings showed opposing effects in the two countries. For example, in the right transverse temporal cortex, cortical thinning was observed in German patients and cortical thickening in Japanese patients.

Even though the analysis of Cook’s distance showed that no single participant had an excessive influence on our results, one participant had an extremely high value for cortical thickness in the right lateral occipital cortex. Thus, the analysis was rerun, excluding the participant in question. The effect became only slightly weaker (*B* = − 0.070 instead of -0.072) and remained statistically significant on the 5%-level.

### Grey matter surface area

**Figure 2:**
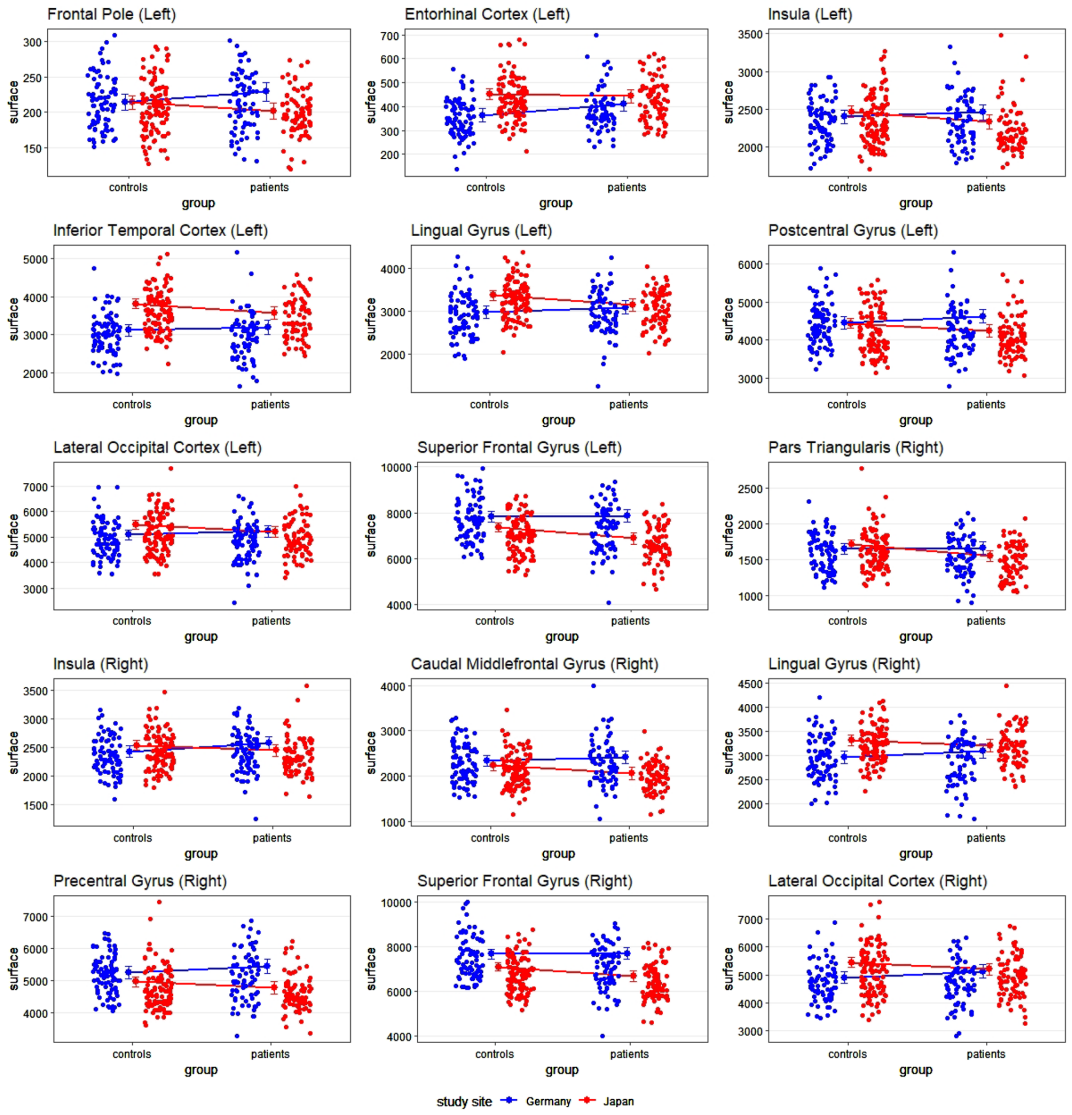
Scatterplots depicting the interaction between group and site with cortical surface area as dependent variable. The small dots represent the individual values per participant; the lines connect the estimated means (i.e., the output of the regression). Error bars indicate the 99% confidence interval.

There were even more areas in which differences in cortical surface area could be identified depending on the interaction of group and site. Interestingly, on a descriptive level, we almost always observed a reduced surface area in the Japanese sample, while there was an increased surface area in the German sample. The statistics of the statistically significant interaction terms are presented in the supplementary material; a graphical depiction is shown in Fig. 2.

### Robustness of results against additional covariates

Following the recommendation of two anonymous reviewers, we aimed at controlling for handedness and years of education, since both have been shown to affect brain structure and are potentially influenced by cultural aspects.

To this end, we re-ran our main analysis (investigating in which areas we could see an interaction between group (schizophrenia vs. controls) and site (Japan vs. Germany) for either cortical thickness or cortical surface area), but this time controlling for years of education and handedness as covariates (in separate analysis). The variables were included in separate analysis to avoid overfitting.

Regarding cortical thickness, results were largely robust against inclusion of handedness and years of education as covariates, only in the right lateral occipital cortex were the results not significant anymore after inclusion of handedness as covariate (see supplementary material).

More changes appeared regarding cortical surface area: results in the left entorhinal cortex and left inferior temporal gyrus, as well as the right lingual, precentral, and superior frontal gyrus were no longer statistically significant after including years of education as covariate (see supplementary material). Additionally, results in the left inferior temporal gyrus and in the right precentral and inferior frontal gyrus were not significant after inclusion of handedness as covariate (see supplementary material).

It should be noted that—representing the real world—right-handers vastly outnumbered left-handers and ambidexters (there were 6% left-handers but 87% right-handers in our sample), so the statistical results should not be over-interpreted.

### Relationship between medication and cortical measures

Because it was previously established that the Japanese patients had a lower use of antipsychotics, we wanted to test whether this could have influenced cortical surface and thickness measurements. Thus, we tested whether CPZ dose was related to cortical thickness and surface area, separated for the German and the Japanese sample (with the same covariates as in the main analysis). There were only two significant findings: higher medication dose was related to larger surface area in the right superior parietal lobule for Japanese patients and with increased cortical thickness in the fusiform gyrus for German patients. Note that in neither area, we previously found significant effects in our main analysis. Thus, there is no indication that medication dose affected the results that we have previously discussed.

### Relationship between duration of illness and cortical measures

Because Japanese patients had a longer average duration of illness, it was tested whether duration of illness had an effect on cortical thickness or cortical surface, separated for the Japanese and German sample (again using the same covariates as in the main analysis). Indeed, some cortical areas were identified in which this was the case. Here, cortical areas are reported in which a significant interaction of group and site has previously been found, as these interaction effects might be confounded by duration of illness. Regarding cortical thickness, there were no statistically significant effects of duration of illness in either of the samples (German patients/Japanese patients). Regarding cortical surface area, there were two statistically significant results for the Japanese samples: longer duration of illness was related to larger surface area in the left precuneus (*B* = 1.22, SE = 0.422, *p*_(adjusted)_ = 0.024) and with reduced surface area in the left transversetemporal cortex (B = − 0.286, SE 0.102, *p*_(adjusted)_ = 0.029). For the German sample, no statistically significant results emerged. Again, in neither area, we previously found significant effects in our main analysis. Thus, there is no indication that duration of illness affected the results that we have previously discussed.

### Relationship between psychopathology and cortical measures

The main focus of this study was to analyze potential differences in cortical surface and thickness between Japanese and German patients with schizophrenia. However, to make full use of the data available, the relationship between psychopathology (as measured with the PANSS) and cortical surface and thickness was investigated in additional analyses. Effects were visible in a number of cortical areas, some of which were areas where interaction effects between group and site have previously been found. For example, positive symptoms were correlated with cortical thickness in the left temporal pole and the right inferior and middle temporal gyrus, as well as with cortical surface area in the bilateral precentral gyrus. Negative symptoms were correlated with cortical thickness in the left paracentral gyrus and cortical surface area in the banks of the superior temporal sulcus. Interestingly, positive symptoms seemed to be correlated with a broader range of cortex areas than negative symptoms.

Additionally, differences in mean PANSS values between the two samples were assessed using t-tests. There was no statistically significant difference between the German and the Japanese sample when we compared the positive symptoms, *t*(153) = − 1.293, *p* = 0.198. Regarding negative symptoms, German patients showed a slightly higher score than Japanese patients (17.7 vs. 15.8), *t*(153) = 2.2939, *p* = 0.023). The full results are shown in the supplementary material**.**

## Discussion

In a large sample of patients with schizophrenia and healthy controls from Japan and Germany, GM thickness and surface area were investigated for potential culture-specific effects of schizophrenia on neural structures. Indeed, both cortical thickness and surface showed pronounced effects of cultural background (Japan vs. Germany) in patients compared to controls.

Regarding cortical thickness, effects were visible in the left precuneus, the left inferior and middle temporal gyrus, right transverse temporal cortex and right lateral occipital cortex, right paracentral lobule and right pars triangularis, showing an increase or decrease of these brain regions. Changes in some of these areas have been reported before in schizophrenia^1,3,8^. For example, visual perception abnormalities in schizophrenia seem to be partly rooted in alterations in the lateral occipital cortex^49,50^, and stimulation of this cortical region might elicit complex visual hallucinations^51^. Most notably, thinning in the (left) inferior and middle temporal gyrus seems to be related to auditory hallucinations^52^. Interestingly, we observed cortical thickening rather than thinning of the inferior and middle temporal gyrus in the German sample, which has previously been associated with psychopathy rather than schizophrenia^53^. However, previous research on British schizophrenia patients also showed increased GM volume in the left temporal lobe of white patients, but not black patients^32^. It should be noted, though, that results in the left inferior middle temporal gyrus were not significant after inclusion of years of education as covariate (see below).

For cortical surface area, effects were even more widespread. We found bilateral effects in the lingual gyrus, the insula and the lateral occipital cortex, as well as effects in the left frontal pole, entorhinal cortex, inferior temporal gyrus and postcentral gyrus, and in the right precentral gyrus, pars triangularis, and superior frontal gyrus. Again, these findings partly match with other findings in previous research. For example, it has long been known that the insula is involved in the core psychotic symptomatology of schizophrenia^54^, e.g. differentiating external and self-generated information, a process that is disturbed in the development of hallucinations. As discussed above, the lateral occipital cortex seems to contribute to the development of visual hallucinations.

While there was no effect of medication on cortical measures, in some areas cortical changes correlated with duration of illness in the Japanese sample. This is at odds with some earlier research indicating that there might not be an effect related to the duration of the illness^10,55–57^, but is in line with research on the toxic brain hypothesis, which did find a connection between duration of illness and brain changes^58,59^.

When controlling for years of education and handedness, a number of brain areas for which we had previously found effects showed no statistically significant results anymore.

When using education as covariate, no statistically significant effects on cortical surface area remained in the left entorhinal cortex and inferior temporal gyrus, as well as the right lingual gyrus, precentral gyrus and superior frontal gyrus. When using handedness as covariate, no statistically significant effects on cortical thickness remained in the right lateral occipital cortex remained, and no statistically significant effects on cortical surface area remained in the left inferior temporal gyrus as well as in the right pars triangularis, and precentral gyrus.

Regarding handedness, it should be noted that only 6% of participants were left-handed (vs. 87% right-handers), and interpretation of results based on such a small group should be done cautiously. Regarding education, it remains unclear whether years of education actually is a confounding variable. It might also be true that cultural differences are reflected in this variable: not only is schooling in general affected by culture, but also because patients with early-onset schizophrenia or prodromal syndrome might be more likely to drop out of school early depending on culture. However, as these are only speculations, these analyses should be interpreted with caution, particularly effects regarding the cortical surface area in the left entorhinal cortex and inferior temporal gyrus, as well as the right lingual gyrus, precentral gyrus and superior frontal gyrus.

We have also tested whether duration of illness affected cortical thickness in either the German or the Japanese sample, since the latter had a longer average duration of illness. No statistically significant effects were found in any of the areas for which we previously found an interaction effect between culture and diagnosis. Thus, it seems unlikely that our results were an artefact produced by differences in duration of illness.

It is noteworthy that many of the brain areas which showed site-specific effects are related to hallucinations in schizophrenia, most notably the insula (which is involved in differentiating external and self-generated information), the inferior and middle temporal cortex (where cortical thickness is inversely correlated with auditory hallucinations^52^) and the lateral occipital cortex (contributing to visual hallucinations^49–51^). Previous behavioral research showed that there are pronounced cultural differences in the prevalence of various kinds of hallucinations^18,19^. Specifically, it has been reported that Asian patients with schizophrenia show more auditory and visual hallucinations than European patients^17^. Investigating whether such differences might correspond with the changes of cortical surface and thickness reported here in patients with schizophrenia, we found that the difference in prevalence might have some relation to the insula: Japanese patients (but not German patients) showed bilaterally reduced surface area in the insula, which might indicate that key functions of the insula, like differentiating self-generated from external information, are disturbed, thereby contributing to hallucinations. These findings are at odds with those of Gong et al.^31^, who showed volume reduction in the (right anterior) insula regardless of ethnicity. However, it might also be that these conflicting results are due to different measurements, as Gong et al. analyzed GM volume, not cortical surface area. Additionally, it might be that the increased cortical thickness of German patients in the left inferior and middle temporal cortex might serve as protective factor against some hallucinations, but this remains speculative.

Another significant result concerns the transverse temporal cortex, which, being a core area of the auditory cortex, has previously been connected to auditory hallucinations^60^. To our knowledge, it is still somewhat unclear whether to expect cortical thickening or thinning in patients with auditory hallucinations^61,62^. Interestingly, both effects are visible in our study: thinning in German patients and thickening in Japanese patients. Although intriguing, whether and how this relates to specificities of auditory hallucinations cannot be determined at this point.

In previous research a lot of focus was put on cortical thinning in schizophrenia. At the same time, cortical thickening has also been reported. For example, cortical thickening in the bilateral anterior temporal lobe has been interpreted as either a compensatory mechanism or a pathological change^10^. Interestingly, in the sample at hand, only the German patients showed cortical thickening in the inferior temporal and the middle temporal gyrus (both of which partly make up the anterior temporal lobe), while the Japanese sample showed no change. Because of methodologically different analyses in previous and the current study it is difficult to determine whether the results reported here completely match those previously reported findings. Still, our results might hint at differences in compensatory mechanism depending on cultural context, especially given the role of the anterior temporal lobe in visual perception^63^. For all areas reported here, however, more research would be needed to establish a clearer link between behavioral manifestations of the disease, its neural correlates and their cultural variation.

Regarding the neural underpinnings of hallucinations, the results in the pars triangularis are particularly interesting, and it might be assumed that there exists a connection to Broca’s area, which is located in the pars triangularis and which has often been shown to be structurally altered in schizophrenia. However, since Broca’s area is typically located in the dominant hemisphere and since the majority of the sample presented here is right-handed, one would expect to see effects in the left hemisphere. As we only observed changes in the right hemisphere, the connection between the findings at hand and Broca’s area becomes somewhat less convincing.

Interestingly, for some of the brain areas in which we found differences between the Japanese and the German sample, differences between “Eastern” and “Western” cultures have been reported previously. For example, differences between healthy Koreans and (white) US-Americans have been reported in the postcentral gyrus, bilateral inferior temporal gyrus, transversetemporal cortex, lingual gyrus, and lateral occipital cortex^29^. Because our statistical analyses take into account the cortical measures of both healthy participants and patients across the two study sites, our results are unlikely to be affected by any baseline differences. Still, the fact that culture-related brain differences regarding schizophrenia are partly represented in areas that show culture-related brain differences in healthy participants seems to warrant further investigation of these areas in future studies.

While the study at hand aimed to investigate cultural influences on neural changes in schizophrenia, it should be noted that this is not equal to contrasting the effects of a uniform “Japanese culture” and a uniform “German culture” on the neural correlates of schizophrenia, as “culture” is difficult to define and “cultural neuroscience” might be perpetuating Western biases^25^. Rather, the variability between culturally divergent samples was investigated, without attributing any potential differences to a monolithic idea of “culture”. Still, one caveat remains: it might be that our study did not only capture cultural effects, but also other site-specific effects—for example, the two sites differ in city size (Kyoto has roughly five times as many inhabitants as Muenster) or in terms of MRI scanners used. Regarding the first point, it should be noted that although differing in size, Muenster and Kyoto do have communalities (e.g., a high percentage of university students, and a historic city center). Regarding the latter point, it seems unlikely that the difference in MRI scanners had an influence on the results, which are based on interaction effects (with healthy controls as baseline at both sites), so effects of MRI scanner would have to be specific to the patient group at one site without affecting the baseline group, or vice versa. This seems rather implausible.

Another potential limitation of our study is the absence of genetic analyses. Indeed, genetic differences might influence the manifestation of the disorder, either directly or indirectly via culture. Disentangling these effects is quite difficult (as one would need a sufficiently large sample of patients with schizophrenia who grew up unaffected by Japanese culture but share the genetics of the Japanese population). There is, however, some evidence that suggests that this might not be a highly relevant aspect: in one study it was shown that Pakistani patients living in Britain show a manifestation of schizophrenia more closely related to White British patients than to Pakistani living in Pakistan^21^. Hence, genetic effects might be less influential than cultural ones. Either way, this issue does not affect one of the central messages of this study, i.e. that the neuroscience of schizophrenia should be using more diverse samples.

From a methodological point of view, it could be criticized that our samples were not exactly matched, e.g. regarding age, medication, duration of illness, etc. In order to maximise our sample size we decided against an exact match and instead controlled for a number of potentially confounding variables and employed cross-validation. Thus, our results likely reflect true differences between both groups. Still, future studies might try to reach similarly large sample sizes in exactly matched patient groups.One might wonder why differences in brain structure and activity occur between humans from different cultures at all, or why culture should influence neural correlates of mental disorders. One prominent factor might be the nature of social interactions and the perception of social events. While earlier concepts (such as a strong divide between collectivistic vs. individualistic cultures) are likely oversimplifying^64,65^, there are still known influences of culture on social relations. For example, there is meta-analytic evidence of differences in neural activation during social tasks in “Western” and “East-Asian” cultures^66^. One interpretation of these results is that “Westerners” show reduced activity in areas related to inferring the mental states of others, but enhanced neural activity in areas related to self-relevance encoding. One might assume that (neural and mental) differences in how people construct themselves and their relationship to others is also highly relevant for mental health, especially for a disorder like schizophrenia that is often characterised by paranoia or delusions related to the behaviour of others, as well as a problems with maintaining self-other boundaries.

On a final note, it is remarkable that the differences we found in GM cortical changes in schizophrenia are already apparent when comparing individuals from two relatively rich, democratic, industrialized countries. It is quite possible that patients from even more diverse countries might also show more differences in the neural correlates of schizophrenia. More research is therefore needed that explicitly investigates differences in patients from other backgrounds. Our findings show the importance of taking cultural background into account when investigating neural correlates of schizophrenia and call for the inclusion of more diverse samples.

## Supplementary Information

Supplementary Information.

## Data Availability

Data are available from the corresponding author upon request.
