# Supplementary material for: Cortical changes in patients with schizophrenia across two ethnic backgrounds
Source: Sci Rep. 2022 Jun 25;12:10810. doi: 10.1038/s41598-022-14914-3 (PMC9233668; doi:10.1038/s41598-022-14914-3)
Supplement: Supplementary file 1 — Supplementary Information. [file 41598_2022_14914_MOESM1_ESM.docx]

# **Supplementary material**

**Supplementary material 1:** Areas with statistically significant interaction effects regarding cortical thickness.

| Cortical region | Estimate of interaction | Standard error | P-value (adjusted) |
| --- | --- | --- | --- |
| *Left* |  |  |  |
| Precuneus | 0.066 | 0.021 | 0.004 |
| Inferior temporal gyrus | -0.109 | 0.034 | 0.004 |
| Middle temporal gyrus | -0.110 | 0.039 | 0.012 |
| *Right* |  |  |  |
| Transverse temporal cortex | 0.127 | 0.040 | 0.005 |
| Paracentral lobule | 0.0710 | 0.027 | 0.024 |
| Inferior frontal gyrus – pars triangularis | -0.0710 | 0.025 | 0.012 |
| Lateral occipital cortex | -0.0720 | 0.030 | 0.039 |
| Medial orbitofrontal cortex | -0.1120 | 0.034 | 0.003 |

**Supplementary material 2:** Areas with statistically significant interaction effects regarding grey matter surface area.

| Cortical region | Estimate of interaction | Standard error | P-value (adjusted) |
| --- | --- | --- | --- |
| *Left* |  |  |  |
| Frontal pole | -26.804 | 9.391 | 0.011 |
| Entorhinal cortex | -58.688 | 22.337 | 0.019 |
| Insula | -197.736 | 74.602 | 0.018 |
| Inferior temporal gyrus | -315.750 | 132.879 | 0.035 |
| Lingual gyrus | -347.868 | 119.186 | 0.009 |
| Postcentral gyrus | -369.781 | 130.956 | 0.012 |
| Lateral occipital cortex | -424.862 | 167.647 | 0.025 |
| Superior frontal gyrus | -533.354 | 200.621 | 0.018 |
| *Right* |  |  |  |
| Inferior frontal gyrus – pars triangularis | -180.044 | 62.630 | 0.010 |
| Insula | -237.500 | 80.649 | 0.008 |
| Caudal middle frontal gyrus | -255.521 | 104.487 | 0.030 |
| Lingual gyrus | -264.877 | 105.559 | 0.026 |
| Precentral gyrus | -372.592 | 159.146 | 0.037 |
| Superior frontal gyrus | -461.575 | 191.834 | 0.032 |
| Lateral occipital cortex | -470.865 | 172.292 | 0.014 |

**Supplementary material 3:** Relationship between duration of illness and grey matter cortical thickness

| Region | Estimate | Standard error | P-value (adjusted) |
| --- | --- | --- | --- |
| *Left* |  |  |  |
| Postcentral gyrus | 0.0007 | 0.0002 | 0.027 |
| Cuneus | 0.0006 | 0.0002 | 0.041 |
| *Right* |  |  |  |
| Medial orbitofrontal cortex | 0.0007 | 0.0002 | 0.000 |

**Supplementary material 4:** Relationship between duration of illness and grey matter surface area

| Region | Estimate | Standard error | P-value (adjusted) |
| --- | --- | --- | --- |
| *Left* |  |  |  |
| Superior frontal cortex | -2.4404 | 0.9183 | 0.0194 |
| *Right* |  |  |  |
| Banks of the superior temporal sulcus | 0.4682 | 0.1450 | 0.0038 |
| Caudal middle frontal gyrus | -1.0243 | 0.4421 | 0.0465 |

**Supplementary material 5**: Relationship between psychopathology and cortical thickness

**Positive Symptoms**

| Cortical region | Estimated effect of PANSS | Standard error | P-value (adjusted) |
| --- | --- | --- | --- |
| *Left* |  |  |  |
| Caudal middle frontal gyrus | -0.005 | 0.002 | 0.043 |
| Temporal pole | 0.0231 | 0.009 | 0.031 |
| *Right* |  |  |  |
| Caudal middle frontal gyrus | -0.005 | 0.002 | 0.031 |
| Entorhinal cortex | 0.017 | 0.007 | 0.041 |
| Inferior temporal gyrus | 0.011 | 0.004 | 0.030 |
| Middle temporal gyrus | 0.011 | 0.004 | 0.031 |
| Postcentral gyrus | -0.005 | 0.002 | 0.033 |
| Supramarginal gyrus | -0.00413 | 0.002 | 0.049 |

**Negative Symptoms**

| Cortical region | Estimated effect of PANSS | Standard error | P-value (adjusted) |
| --- | --- | --- | --- |
| *Left* |  |  |  |
| Paracentral gyrus | -0.005 | 0.002 | 0.043 |

**Supplementary material 6**: Relationship between psychopathology and cortical surface area

**Positive Symptoms**

| Cortical region | Estimated effect of PANSS | Standard error | P-value (adjusted) |
| --- | --- | --- | --- |
| *Left* |  |  |  |
| Precentral gyrus | -26.4 | 9.98 | 0.02 |
| *Right* |  |  |  |
| Caudal middle frontal gyrus | -21.4 | 6.93 | 0.006 |
| Precentral gyrus | -26.3 | 11.3 | 0.045 |

**Negative Symptoms:**

| Cortical region | Estimated effect of PANSS | Standard error | P-value (adjusted) |
| --- | --- | --- | --- |
| *Right* |  |  |  |
| Banks of the superior temporal sulcus | 4.63 | 2.02 | 0.045 |

**Supplementary material 7:** Areas with statistically significant interaction effects regarding cortical thickness, with education as covariate

| Cortical region | Estimate of interaction | Standard error | P-value (adjusted) |
| --- | --- | --- | --- |
| *Left* |  |  |  |
| Precuneus | 0.066 | 0.021 | 0.006 |
| Inferior temporal gyrus | -0.110 | 0.035 | 0.006 |
| Middle temporal gyrus | -0.108 | 0.040 | 0.0202 |
| *Right* |  |  |  |
| Transverse temporal cortex | 0.137 | 0.041 | 0.003 |
| Paracentral lobule | 0.065 | 0.027 | 0.041 |
| Inferior frontal gyrus – pars triangularis | -0.072 | 0.023 | 0.008 |
| Lateral occipital cortex | -0.071 | 0.029 | 0.044 |
| Medial orbitofrontal cortex | -0.109 | 0.034 | 0.006 |

**Supplementary material 8:** Areas with statistically significant interaction effects regarding grey matter surface area, with education as covariate

| Cortical region | Estimate of interaction | Standard error | P-value (adjusted) |
| --- | --- | --- | --- |
| *Left* |  |  |  |
| Frontal pole | -29.382 | 9.642 | 0.007 |
| Insula | -193.159 | 76.78 | 0.030 |
| Lingual gyrus | -324.720 | 120.323 | 0.020 |
| Postcentral gyrus | -333.474 | 133.665 | 0.032 |
| Lateral occipital cortex | -392.326 | 170.852 | 0.0496 |
| Superior frontal gyrus | -483.336 | 205.639 | 0.044 |
| *Right* |  |  |  |
| Inferior frontal gyrus – pars triangularis | -165.13 | 63.767 | 0.02456 |
| Insula | -206.484 | 82.211 | 0.02982 |
| Caudal middle frontal gyrus | -245.896 | 107.144 | 0.04946 |
| Lateral occipital cortex | -422.389 | 173.206 | 0.03582 |

**Supplementary material 9:** Areas with statistically significant interaction effects regarding cortical thickness, with handedness as covariate

| Cortical region | Estimate of interaction | Standard error | P-value (adjusted) |
| --- | --- | --- | --- |
| *Left* |  |  |  |
| Precuneus | 0.063 | .021 | 0.013 |
| Inferior temporal gyrus | -0.109 | .035 | 0.008 |
| Middle temporal gyrus | -0.112 | .040 | 0.020 |
| *Right* |  |  |  |
| Transverse temporal cortex | 0.126 | .041 | 0.009 |
| Paracentral lobule | 0.076 | .027 | 0.020 |
| Inferior frontal gyrus – pars triangularis | -0.071 | .023 | 0.012 |
| Medial orbitofrontal cortex | -.098 | .035 | 0.021 |

**Supplementary material 10:** Areas with statistically significant interaction effects regarding grey matter surface area, with handedness as covariate

| Cortical region | Estimate of interaction | Standard error | P-value (adjusted) |
| --- | --- | --- | --- |
| *Left* |  |  |  |
| Frontal pole | -28.676 | 9.749 | 0.013 |
| Entorhinal cortex | -60.729 | 22.726 | 0.026 |
| Insula | -211.646 | 76.941 | 0.021 |
| Lingual gyrus | -399.864 | 121.835 | 0.019 |
| Postcentral gyrus | -361.265 | 135.866 | 0.027 |
| Lateral occipital cortex | -427.245 | 174.438 | 0.045 |
| Superior frontal gyrus | -532.007 | 208.161 | 0.035 |
| *Right* |  |  |  |
| Insula | -43.989 | 83.233 | 0.012 |
| Caudal middle frontal gyrus | -290.195 | 107.142 | 0.022 |
| Lingual gyrus | -260.195 | 108.261 | 0.048 |
| Superior frontal gyrus | -484.107 | 198.796 | 0.045 |
| Lateral occipital cortex | -427.586 | 174.848 | 0.044 |

**Supplementary material 11: Diagnosis by nationality**

| **ICD-10-Diagnosis** | **German** | **Japanese** |
| --- | --- | --- |
| Paranoid schizophrenia (F20.0) | 40 | 27 |
| Disorganized schizophrenia (F20.1) | 6 | 3 |
| Catatonic schizophrenia (F20.2) | 1 | 2 |
| Undifferentiated schizophrenia (F20.3) | 8 | 2 |
| Schizoaffective disorder, mixed type (F25.2) | 1 | 0 |
| Acute polymorphic psychotic disorder with symptoms of schizophrenia (F23.1) | 1 | 1 |
| Residual schizophrenia (F20.5) | 16 | 0 |
| Schizoaffective Disorder, manic type (F25.0) | 1 | 1 |
| No data | 0 | 45 |
